# The British Journals: Midwifery

**Published:** 1839-01

**Authors:** 


### MIDWIFERY.

*Researches into the History of the Umbilical Cord in the Human Subject.* By F. Churchill, m.d., Physician to the Western Lying-in Hospital, Dublin.

[This is a very elaborate and valuable paper, comprising, as the author truly states, all the facts on record which bear upon the history of the umbilical cord. It is so condensed as to be unsusceptible of abridgment, and is too long for extraction. We select that portion of the section devoted to *Prolapsus of the Cord,* which gives the statistics of its frequency:]

The cord may be prolapsed either at the commencement or during labour. Though by no means of unfrequent occurrence, its comparative frequency varies very much.

Mad. Boivin mentions its occurrence 38 times, (25 times before and 13 during labour,) out of 20,517 cases. Twenty-five cases were turned, and in 13 the forceps were used. Twenty-nine children were saved, and seven lost; two were putrid.

Mad. La Chapelle met with 23 cases out of 15,652: 13 were treated by the forceps, and 10 by turning: 17 were saved, and 6 lost. M. Baudelocque, out of 17,499 labours, reports 41 cases of prolapsed funis. Dr. Bland gives one case of prolapse in 1897 cases of labour.

Dr. Merriman gives 11 cases in 2947 labours. Dr. Granville, one case in 640 labours.

Richter, of Moscow, found 4 in 624; and Mazzoni, 18 in 450 cases. Dr. Clarke, in his Report of the Dublin Lying-in Hospital, met with 66 cases of prolapsus of the cord in 10,387 deliveries; but the doctor does not believe that all the cases of this kind were recorded in the registry. Seventeen children were born alive.

Dr. Collins, in his valuable report of 16,654 cases, occurring in the same hospital between the years 1826 and 1833, states that 97 cases of prolapsed funis occurred, 12 of them in twin cases (i. e. I presume the cord of one of the twins prolapsed): 24 children were saved, 7 were putrid.

At the Coombe Lying-in Hospital in this city, Mr. Gregory reported, in 1830, that, since its commencement in February 1829, 691 patients had been delivered, among which there were seven funis presentations, four of which were lost.

At the Wellesley Dispensary, Dr. Samuel Cusack reported, in 1830, five cases of prolapse of the funis in 398 labours; and all appear to have been lost.

In the reports of this institution for the year 1832, 1833, by Dr. Maunsell, there were two cases of prolapse of the funis in 839 labours. No mention is made of the result to the children.

In the Western Lying-in Hospital, 31, Arran Quay, Dublin, between November 1, 1835, and December 31, 1837, there were 616 women delivered, and two cases of prolapsed funis. Both children were lost.

Dr. Beatty reports that six cases of prolapse out of 1182 labours occurred at the New Lying-in Hospital, between April 1834, and August 31st, 1837. Four of the children were lost.

If we add these together, they amount to 90,983 deliveries, and 322 cases of prolapse of the cord, or one in every 282½.

*Edinburgh Med. and Surg. Journal. October,* 1838.

